# Application of intranasal dexmedetomidine in magnetic resonance imaging of preterm infants: The ED50, efficacy and safety analysis

**DOI:** 10.1097/MD.0000000000038040

**Published:** 2024-05-03

**Authors:** Shengjun Wan, Wei Wu, Wenhao Bu

**Affiliations:** a Department of Anesthesiology, Maternal and Child Health Hospital of Hubei Province, Tongji Medical College, Huazhong University of Science and Technology, Wuhan, China; b Department of Anesthesiology, CR & WISCO General Hospital, Affiliated to Wuhan University of Science and Technology, Wuhan, China.

**Keywords:** dexmedetomidine, magnetic resonance imaging, sedation

## Abstract

**Background:**

Infants undergoing magnetic resonance imaging (MRI) often require pharmacological sedation. Dexmedetomidine serves as a novel sedative agent that induces a unique unconsciousness similar to natural sleep, and therefore has currently been used as the first choice for sedation in infants and young children.

**Objective:**

To determine the 50% effective dose (ED50) and 95% confidence interval (95%CI) of intranasal dexmedetomidine for MRI in preterm and term infants, and to observe the incidence of adverse events. To explore whether there were differences in ED50 and 95%CI, heart rate (HR) and blood oxygen saturation (SpO_2_), the induction time and wake-up time and the incidence of adverse events between the 2 groups, so as to provide guidance for clinical safe medication for the meanwhile.

**Methods:**

A total of 68 infants were prospectively recruited for MRI examination under drug sedation (1 week ≤ age ≤ 23 weeks or weight ≤ 5kg). The children were divided into 2 groups according to whether they had preterm birth experience (Preterm group, Atterm group). The Dixon up-and-down method was used to explore ED50. The basic vital signs of the 2 groups were recorded, and the heart rate and SpO_2_ were recorded every 5 minutes until the infants were discharged from the hospital. The induction time, wake-up time and adverse events were recorded.

**Results:**

The ED50 (95%CI) of intranasal dexmedetomidine in the Preterm group and the Atterm group were 2.23 (2.03–2.66) μg/kg and 2.64 (2.49–2.83) μg/kg, respectively (*P* < .05). the wake-up time was longer in Preterm group (98.00min) than in Atterm group (81.00 min) (*P* < .05), the incidence of bradycardia in Preterm group was 3/33, which was higher than that in Atterm group (1/35). There was no difference in the induction time between the 2 groups (*P* > .05), and there was no significant difference in other adverse events.

**Conclusions:**

Intranasal dexmedetomidine can be safely used for sedation in preterm infants undergoing MRI. Compared with term infants, preterm infants have a lower dose of dexmedetomidine, a higher incidence of bradycardia, and a longer weak-up time.

## 1. Introduction

Magnetic resonance imaging (MRI) is one of the most common cranial examinations in infants and young children.^[1]^ However, during the acquisition and scanning process, the patient is required to stay still for a long time under a high level of noise from the equipment. Any movement during this time degrades and distorts the images. Such as during abdominal and ocular MRI examinations, small movements like irregular breathing and eye rotation will affect image acquisition,^[2,3]^ so it is often difficult for infants and young children to cooperate.^[4]^ In order to eliminate children fear and ensure the quality of imaging, it is often necessary to use some sedatives before MRI examination.

Dexmedetomidine, as a highly selective α_2_ adrenoceptor agonist-mediated by an inhibitory effect on locus coeruleus noradrenergic neurons,^[5]^ is serve as a novel sedative to induce an arouse state of sedation similar to natural sleep.^[6]^ In addition to intravenous injection, dexmedetomidine can also be administered through a variety of noninvasive routes such as intranasal, mucosal, and oral administration. Among them, intranasal dexmedetomidine as a noninvasive and painless method of administration is most commonly accepted by children and their families.^[7,8]^ Meanwhile, this administration can achieve a high success,^[9]^ because the drug absorbed through the mucosa could avoid the first-pass metabolism of the liver,^[10]^ and with a high bioavailability (93%) it had been reported that intranasal administration of 3–4 μg/kg could ensure a good sedation with low incidence of adverse events.^[11]^ Olgun et al utilized intranasal administration of 4 μg/kg dexmedetomidine in infants with an average age of 7 months, and the success rate of MRI sedation was 94.2%.^[12]^ Therefore, Dexmedetomidine has been shown to be less neurotoxic or potentially neuroprotective than other sedatives, and is now widely used for sedation before MRI in infants and young children.^[13–15]^

In fact, preterm infants account for a substantial proportion of the population of infants undergoing MRI, with an increasing number of babies being born before 37 weeks of gestation due to rising rates of preterm birth. Premature babies needs more carefulness when pharmacological sedation, since they often have complex medical problems including neurological dysfunction, breathing problems and developmental delays.^[16]^ Havidich et al found that preterm infants, especially those of low birth weight and younger age, were at increased risk for sedation-related adverse events, such as airway obstruction, cough, and desaturation, and that the incidence of sedation-related adverse events among preterm infants was as approximately twice high as among term infants.^[17]^ Therefore, sedation management is very important for infants, especially premature infants undergoing MRI.

Most of the clinical sedation studies at this stage are for older children, and there is still a lack of studies of preterm infants on the clinically safe dose range, adverse effects, and the comparison with term infants of intranasal dexmedetomidine for MRI sedation. Hence, in this study, with hypothesis of the different the 50% effective dose (ED50) of intranasal dexmedetomidine sedation between the preterm infants and term infants, we apply the Dixon up-and-down method to explore the ED50 and observe the adverse events during sedation. And finally, we acquire the ED50 and 95%CI of intranasal dexmedetomidine for MRI sedation in preterm and term infants, as well as the incidence of adverse events. Additionally, with exploring whether there are differences in ED50 and 95%CI, HR and blood oxygen saturation (SpO_2_), the induction time and wake-up time and the incidence of adverse events between the 2 groups, we will provide a guidance for clinical safe medication.

## 2. Materials and methods

### 2.1. Subjects

The Dixon up-and-down method was utilized to design this prospective study. The ethical approval was obtained from Research Ethics Committee of Maternal and Child Health Hospital of Hubei Province on April 10, 2023. Infants who underwent MRI examination in the sedation center of our hospital within 3 months from April 20, 2023 to July 20, 2023 were collected. Written informed consent was obtained from a parent or legal guardian for all children recruited for the study. All the children were divided into 2 groups according to whether they had preterm birth experience: Preterm group and Atterm group. Infants in preterm group had a gestational age of <37 weeks. Infants in Atterm group were born after 37 weeks of gestation. In the process of recording the age of preterm infants, we added a definition as corrected age. We defined the corrected age of preterm infants as the sum of gestational age in weeks and postnatal age in weeks after birth minus 37 weeks. We define the age of term infants in weeks after birth. Meanwhile, we ignored days shorter than a week in the age recording process. Later in this article, when we refer to the age of preterm infants, we mean the age after correction. All the children were accompanied by their family members during the whole process of the experiment. Inclusion criteria were as follows: Infants with ASA class I to III (1 week ≤ age ≤ 23 weeks or weight ≤ 5kg) who were scheduled for MRI under intranasal dexmedetomidine in our sedation center were enrolled, regardless of gender. The exclusion criteria were allergy to dexmedetomidine, nasal structure abnormality, bradycardia, hepatic and renal dysfunction, significant developmental delay or behavioral problems, and use of β-blockers or other sedatives before the examination.

### 2.2. Methods

#### 2.2.1. Sedation

Infants were fasted from formula milk for 2 hours, breast milk for 1 hour, and sleep deprivation for more than 2 hours as required. Parents should accompany their children into the MRI room 20 to 30 minutes before the examination to familiarize themselves with the environment. HR and SpO_2_ were monitored using a special MRI monitor (Patient Monitor, Tesla DUO, MIPM Mammendorfer Institut fuer Physik und Medizin GmbH), emergency equipment and medicine were prepared in advance to deal with emergencies. The needle stem at the front end of the 1 mL syringe needle plug was removed with a vascular clamp to facilitate deep intranasal administration. The nostrils of all infants were carefully cleaned. Undiluted dexmedetomidine was administered slowly into both nostrils of each child by an anesthetist and the ala of their noses was gently rubbed, with the child lying in the supine position for 1 minute. The sedation effect was evaluated every 5 minutes by the nurses who were unaware of the drug dose, and the evaluation was based on the MOAA/S sedation score. Sedation efficacy was assessed as follows: effective sedation was defined as a MOAA/S score of ≤ 3 points in 30 minutes after administration. Failed sedation was defined as MOAA/S score > 3 points at 30 minutes after administration or inability to complete MRI scan due to body movement. After successful sedation, the child was placed on the scanning table in the MRI room, earplugs were used to prevent noise, scanning at this time results in an image free of motion artifacts. After the examination, the infant continued to be observed until fully awake.

#### 2.2.2. Dose management

The initial dose of intranasal dexmedetomidine was set at 2 μg/kg, according to Dixon up-and-down method, this dose was decrease (or increased) by 0.25 μg/kg, depending on the successful (or unsuccessful) result of previous patient. Taking “sedation failure-sedation success” as the inflection point, and going on until 7 inflection points appeared, the experiment was completed. When sedation was not effective, an anesthesiologist administered a rescue dose of 0.5 μg/kg dexmedetomidine to ensure achieve an adequate depth of sedation, such children were considered as failed sedation. The rescue dose was set based on our experience with infant sedation in this center. Children who failed after an additional intranasal infusion of dexmedetomidine were switched to other sedation methods or were given sedation at another time.

#### 2.2.3. Monitoring and observation

Heart rate and SpO_2_ were recorded at baseline and every 5 minutes until the infants awake. Infant did not receive oxygen during the MRI. The induction time of sedation, adverse events such as reflux and vomiting, bradycardia and respiratory depression were recorded. Bradycardia was defined as a 20% decrease in heart rate from the previous standard level, and respiratory depression was defined as SpO_2_ < 90%. The induction time of sedation was defined as the time from the beginning of drug administration to the attainment of a satisfactory sedative effect. All observations and data collection were performed by a nurse who was blinded to the drug dosage.

#### 2.2.4. Discharge criteria

After completion of the examination, the child was transferred to the recovery room for continued observation until discharge criteria were met. The discharge criterion was Steward awakening score ≥ 5, and the weak-up time was defined as the time from successful sedation to discharge criteria.

### 2.3. Statistical analysis

Statistical analysis was performed using SPSS version 26.0. The probit regression was used to obtain ED50 and 95%CI. The Shapiro–Wilk test was used to test the normality of quantitative data. Quantitative data with normal distribution were expressed as the mean (standard deviation [SD]). Nonnormally distributed data were presented as median (interquartile range [IQR]). The Wilcoxon signed-rank sum test was used to compare the ED50 between groups. The Mann–Whitney U test was used to compare the age, weight, fasting time, wake-up time between the 2 groups. The Chi-square test was used to compare the gender between the 2 groups. The Wilcoxon signed-rank sum test was used to compare HR and SpO_2_ between the 2 groups. Independent sample t test was used to compare the onset time between the 2 groups. *P* < .05 was considered statistically significant.

## 3. Results

### 3.1. General data

A total of 68 infants were enrolled in this study, including 33 in the Preterm group and 35 in the Atterm group. There were no significant differences in age, weight, fasting time, and gender between the 2 groups (*P* > .05). The data are presented in Table 1.

**Table 1:** Demographic data of the patients in the 2 groups.

	Preterm group (33)	Atterm group (35)	*P*
Age (wk)	Corrected age	Postnatal age	
	16.00 (4.50)	16.97 (3.11)	.60
Weight (kg)	4.97 (0.36)	5.10 (0.40)	.06
Fasting time (h)	1.30 (0.30)	1.40 (0.30)	.80
Male/female	17/16	13/22	.33

Data are presented as mean (SD), median (IQR), or number (n/n).

### 3.2. Dose-response to intranasal dexmedetomidine

**Figure 1.**
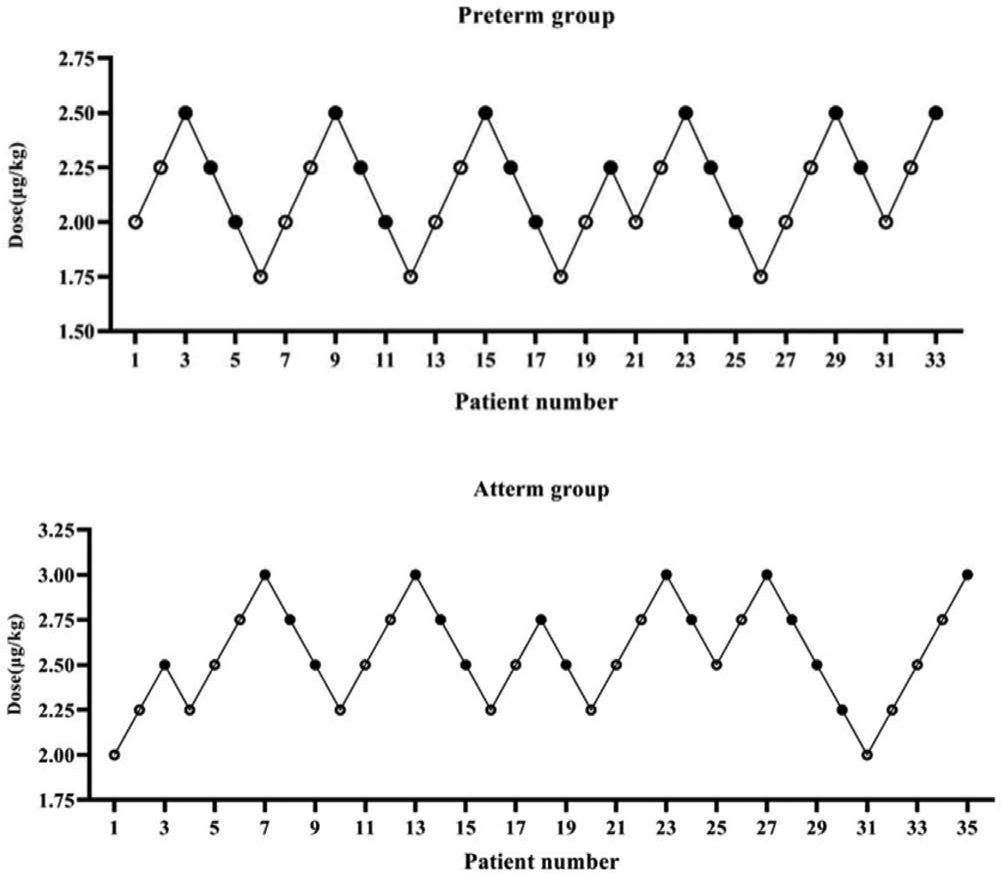
Assessment of success or failure of sedation with a single dose of intranasal dexmedetomidine in patients. Data were recorded for the statistical analysis with Dixon up-and-down method. The solid circles represent instances of successful sedation and the open circles represent instances of failed sedation.

The sequence of the dose-response data for each group is shown in Figure 1. Using Dixon up-and-down method, we found the ED 50 (95% CI) values of intranasally administered dexmedetomidine was 2.23 (2.03–2.66) μg/kg and 2.64 (2.49–2.83) μg/kg in the Preterm group and Atterm group, respectively. The ED 50 of the Preterm group was significantly lower than those of the Atterm group (*P* < .05).

### 3.3. Hemodynamics

The hemodynamics of the patients are shown in Table 2, Dexmedetomidine administration resulted in the heart rate of infants in the 2 groups being significantly lower than at baseline (*P* < .05), the bradycardia were 9.09% and 2.86% for Preterm group and Atterm group, respectively. Bradycardia occurred in 3 of 33 preterm infants, and 1 of these required administration with atropine. Bradycardia occurred in 1 of 35 term infants but did not require atropine treatment. None of the infants showed respiratory depression, which oxygen saturation drop below 90%. But we did observe the occurrence of altered respiratory movements in sedated infants, of these, 4 were in the preterm group and one in the Atterm group. Presented as intermittent deep wheezing breathing. There was no difference in the induction time between the 2 groups. The weak-up time was longer in Preterm group than in Atterm group (98.00 minutes vs 81.00 minutes) (*P* < .05). We observed no reflux or vomiting in either group. Besides, white lips were observed in 3 preterm infants and one term infant.

**Table 2:** Intra-group comparison of HR, SpO2, comparison of Induction time and weak-up time in the 2 groups.

	Preterm group (33)	Atterm group (35)
HR(/min)		
T0	142.88 (5.78)*	140.74 (5.03)†
T1	127.12 (8.87)*	126.97 (6.26)†
Bradycardia (%)	3/33 (9.09%)	1/35 (2.86%)
SpO_2_(%)		
T0	98.00 (1.00)‡	98.00 (1.00)§
T1	98.00 (1.00)‡	98.00 (1.00)§
Induction time (min)∥	18.91 (2.58)	18.69 (2.35)
Wake-up time (min)¶	98.00 (3.50)	81.00 (17.00)
White lips (%)	3 (9.09%)	1 (2.86%)
Atropine administration (%)	1 (3.03)	0 (0)
Breathing change (%)	4 (12.12%)	1 (2.86%)

Data are presented as Mean (SD), median (IQR).

T0: Baseline T1: After admission to the operating room.

HR between the T0 and T1.

* Preterm group, *P* < .05.

† Atterm group, *P* < .05.

SpO_2_ between the T0 and T1.

‡ Preterm group, *P* > .05.

§ Atterm group, *P* > .05.

Induction time between the Preterm group and Atterm group.

∥ *P* > .05.

Wake-up time between the Preterm group and Atterm group.

¶ *P* < .05.

## 4. Discussion

There are more studies on MRI sedation in children, however, studies on MRI sedation in preterm and term infants are relatively less. This study examined that the dosage in preterm infants is lower than that in term infants, by using a small sample size to determine the effective dose of intranasal dexmedetomidine for MRI sedation in preterm and term infants using Dixon up-and-down method. At the same time, we find that preterm infants have a higher proportion of lower heart rate. There is no obvious decrease in oxygen saturation and other adverse events in the 2 groups. Therefore, this study confirms the safety of this administration method and dose for preterm and term infants undergoing MRI sedation.

Nicolas et al investigated the safety of intravenous dexmedetomidine alone for procedural sedation for MRI in preterm and term infants.^[13]^ But, it is difficult for infants to establish intravenous access, moreover, the establishment of intravenous access for MRI examination not only increases the difficulty of clinical work, but also increases the psychological burden of family members. Therefore, in this study, an alternative route of intranasal dexmedetomidine was used, which was more acceptable to parents because of its noninvasive and painless operation. Tug et al used intranasal dexmedetomidine of 3 μg/kg and 4μg/kg for sedation in 60 children aged 1 to 10 years undergoing MRI examination, and found that 4 μg/kg was more likely to achieve effective sedation.^[11]^ In the study by Lin Qiu et al, the initial intranasal dexmedetomidine dose was 2 μg/kg, and the ED50(95%CI) of the final intranasal dexmedetomidine sedation dose was 3.1 (2.8–3.3).^[18]^ The initial dose set for this study was also 2 μg/kg, the resulting ED50(95%CI) were 2.23 (2.03–2.66) μg/kg and 2.64 (2.49–2.83) μg/kg, respectively. There may be 3 reasons for the lower dose compared with the results of the above studies: First of all, the infants in this study underwent at least 2 hours of sleep deprivation before sedation. It is well known that younger toddlers need more sleep per day. Newborns sleep for up to 80% of the day, while most toddlers and preschoolers sleep for half or more of the day.^[19]^ Infants who have undergone sleep deprivation fall asleep more easily when the disturbance is stopped, allowing them to achieve a sufficiently deep sleep with a lower dose; secondly, the subjects in this study were younger in age than those in the previous study. Pott et al demonstrated reduced dexmedetomidine clearance in infants and young children compared with older children.^[20]^ The reduced clearance resulted in a longer duration of drug action, so the lower dose provided adequate depth of sedation for infants in our study. This is consistent with the conclusions of a study on differences in the dose of chloral hydrate used for sedation in children of different ages,^[21]^ which showed that the dose of chloral hydrate used for sedation was lower in newborns than in infants, and the dose was lower in infants than in children older than 1 year of age; finally, in this experiment, infants fasted for no more than 2 hours. Hunger could cause babies to cry and struggle to sleep and this problem was avoided by a shorter fasting period.

Our study shows that in preterm and term infants with postconceptional 1 week ≤ age ≤ 23 weeks or weight ≤ 5kg, the ED50(95%CI) of intranasal dexmedetomidine is 2.23 (2.03–2.66) μg/kg and 2.64 (2.49–2.83) μg/kg, respectively. The ED50 of preterm infants was significantly lower than that of full-term infants, this might be related to the immature development of organs such as liver and brain in preterm infants.^[22]^ As the largest solid organ in the human body, the liver is responsible for many important functions, including the production of plasma proteins and the biotransformation of exogenous substances and endogenous metabolites. Before birth, the fetus mainly depends on the mother liver function, even after birth, the newborn liver function still has a certain degree of immaturity. Compared with term infants, preterm infants were more likely to develop complications of immature liver function, such as decreased plasma protein production and impaired drug metabolism.^[23]^ On the one hand, decreased plasma protein levels, including albumin, total protein, and α-1-acid glycoprotein, reduced the ability of plasma proteins to bind drugs^[24]^; on the other hand, premature infants have a weak capacity for drug clearance metabolism compared with term infants due to the immaturity of hepatic glucuronidation. Both factors contribute to higher concentrations of free dexmedetomidine which cross the blood-brain barrier and bind to locus coeruleus noradrenergic neuronal receptors to produce sedation in preterm infants.^[25]^ The study by Mikkelsen et al also found that the plasma morphine elimination rate of full-term infants was lower than that of preterm infants, which may be due to the immaturity of hepatic glucuronidation,^[26]^ further supporting our speculation.

In the present study we monitored HR and SpO_2_ of infants. The heart rate decreased significantly in both groups, and bradycardia occurred in 3 cases in the premature group, one of whom needed atropine to correct. Bradycardia occurred in one case in the Atterm group. The mechanism of its negative chronotropic effect may be related to the central sympatholytic effect of dexmedetomidine, and its parasympathetic effect induces calcium ion transmembrane transport in cardiomyocytes.^[27,28]^ Investigations have shown that dexmedetomidine-induced bradycardia should be considered a predictable physiological response to dexmedetomidine and that most of these heart rate-related responses are not clinically significant.^[29]^ A study by Mason, K P et al found when decreased HR occurred during intranasal dexmedetomidine administration, they did not detect any accompanying blood pressure changes.^[30]^ In a meta-analysis, no children who received intranasal dexmedetomidine as premedication had fluctuations in blood pressure that required treatment.^[31]^ Because intranasal dexmedetomidine had little effect on blood pressure, in this study, we avoided stimulating the children not to perform blood pressure measurements during MRI scans. The study by Jeana E et al found that preterm infants were more likely to develop airway problems during MRI sedation.^[17]^ In this study, we found no effect of intranasal dexmedetomidine on SpO_2_ in preterm and term infants, this might be due to the fact that we did not monitor SpO_2_ throughout the examination and our criteria for oxygen saturation reduction. But we did observe the occurrence of altered respiratory movements in sedated children, of these, 4 were in the preterm group and one in the Atterm group. Presented as intermittent deep wheezing breathing, but oxygen saturation did not drop below 90%, so ultimately it not thought to have an effect on SpO_2_, which was consistent with the study by Nicolas Leister.^[13]^ A study of 800 children examined under sedation by Qu et al found that preterm infants were more likely to experience nausea, vomiting and delayed awakening.^[32]^ None of the infants in this study experienced nausea and vomiting, and the term infants were significantly more likely to be discharged home than premature infants, which was consistent with the above findings. The results showed that intranasal dexmedetomidine has a greater impact on heart rate when used for sedation in preterm and term infants. The preterm infants were more likely to experience altered breathing and delayed awakening.

Our study also have several limitations. First, the ED50 is obtained by Dixon up-and-down method, which allows a small sample size, but a more accurate value is acquired from a larger sample size study and from a single center, which makes a difficult generalization. Second, we do not evaluate the sleep quality of the children on the previous day. The different degrees of sleep deprivation in children may have some interference with our study. Third, we do not monitor the children heart rate, SpO_2_, and blood pressure throughout the study, which may cause some limitations in our final results.

Despite these limitations, this study helps to determine the ED50 of intranasal dexmedetomidine for sedation in full-term and preterm infants for MRI examination. Meanwhile, it demonstrates the possible risks of these 2 types of children during this process, which can provide guidance for clinical practice.

## Acknowledgments

We sincerely thank all our research participants who contributed time and effort to this research.

## Author contributions

**Data curation:** Shengjun Wan, Wei Wu.

**Formal analysis:** Shengjun Wan.

**Investigation:** Wei Wu.

**Supervision:** Wenhao Bu.

**Visualization:** Wenhao Bu.

**Writing – original draft:** Shengjun Wan, Wei Wu.

**Writing – review & editing:** Wenhao Bu.
